# Distribution and Abundance of Phlebotominae, Vectors of Leishmaniasis, in Argentina: Spatial and Temporal Analysis at Different Scales

**DOI:** 10.1155/2012/652803

**Published:** 2012-01-19

**Authors:** María Gabriela Quintana, María Soledad Fernández, Oscar Daniel Salomón

**Affiliations:** ^1^Instituto Superior de Entomología, FCN, Universidad Nacional de Tucumán-Consejo Nacional de Investigaciones Científicas y Técnicas (CONICET), Miguel Lillo 205, T4000 JFE San Miguel de Tucumán, Argentina; ^2^Centro Nacional de Diagnóstico e Investigación en Endemo-epidemias, CONICET, Paseo Colón 568, 1er Piso, C1063 ACS Ciudad de Buenos Aires, Argentina; ^3^Instituto Nacional de Medicina Tropical, CONICET, Jujuy y Neuquén, N3370 BPD Puerto Iguazú, Argentina

## Abstract

The spatial-temporal analysis of the abundance of insects, vectors of tegumentary leishmaniasis (TL) and visceral leishmaniasis (VL), was performed in Argentina using spatial-temporal increasing scales. In the microscale (microfocal), the effect of the primary vegetation-crop interface in vector abundance was observed, and also how the shelters, food sources, and other environmental characteristics contribute to habitat microheterogeneity and so to a microheterogeneous vector distribution. In the mesoscale (locality or epidemic focus), the results from different foci of TL (rural and periurban) and VL (urban) suggested a metapopulation structure determined partially by quantifiable habitat variables that could explain the increase of risk associated to an increase of vector-human contact due to climatic or anthropogenic changes. In the macroscale (regional), captures of vectors and records of human cases allowed the construction of risk maps and predictive models of vector distribution. In conclusion, in order to obtain valid results transferrable to control programs from spatial studies, special attention should be paid in order to assure the consistency between the spatial scales of the hypotheses, data, and analytical tools of each experimental or descriptive design.

## 1. Introduction

The leishmaniases comprise a set of clinical manifestations produced by different Trypanosomatidae parasites of the genus *Leishmania*, transmitted by the bite of the female of Phlebotominae, in America from the genus *Lutzomyia*. The three main clinical forms are visceral leishmaniasis (VL), cutaneous leishmaniasis (CL), and mucocutaneous leishmaniasis (ML), the last two are called together tegumentary leishmaniasis (TL).

In Argentina were characterized four species of *Leishmania* from human cases: *Leishmania* (*Viannia*) *braziliensis*, *Le*. (*Leishmania*) *amazonensis*, *Le*. (*Viannia*) *guyanensis*, associated with cutaneous leishmaniasis, and *Le. infantum* associated to visceral leishmaniasis [1].

The association of the parasite *Leishmania* with its vector is generally “species specific,” with restrictive vector competence, although permissive vector species were also described [2, 3]. *Lutzomyia* is the largest genus of vectors present in America, with approximately 500 species, 40 of them are vectors of leishmaniases. The classification adopted in this study was from Young and Duncan [4]. 

In Argentina, 28 species of Phlebotominae (23 species of *Lutzomyia*, 4 of *Brumptomyia,* and 1 species of *Oligodontomyia*) were recorded, distributed in 13 provinces, and from those were incriminated as vectors of TL caused by *Le. braziliensis*: *Lutzomyia neivai*, *Lu. whitmani*, *Lu. cortelezzii complex *(*Lu. Cortelezzii–Lu. sallesi*), *Lu. Migonei,* and *Lu. Pessoai,* the first three being reported with natural infection of *Leishmania* or *Le. braziliensis*. The vector incriminated in VL caused by *Le. infantum* was *Lu. longipalpis* [1].

In Argentina, the reservoir of *Le. braziliensis* has not been identified yet, and no animal meets all the criteria required to be defined as such, but horses, cats, canids, and primates can become infected and have clinical manifestations [5, 6]. The dog, although highly susceptible, seems not likely to be a reservoir for transmission of *Le. braziliensis* to humans, given the low supply of parasites to the vector, although this topic has been thoroughly discussed without conclusive results yet [5, 7, 8]. By contrast, the dog was incriminated as the main reservoir of the VL in urban foci [1].

The knowledge of the mechanisms that modulates the incidence of vector-borne diseases due to changes in the environment can contribute to the planning of control strategies [1, 9, 10]. This approach is especially important for TL, as the distribution and abundance of vectors are usually the best indicators in space and time of the parasite transmission, while the analysis based on human cases could introduce errors and bias both in space and time by the asymptomatic unrecorded incidence and the dispersion of the data obtained by anamnesis, respectively [1, 10, 11]. The predictive risk maps in turn allow a proper allocation of resources and to prioritize vector control activities.

However, in this kind of spatial analysis, the outcome of any study will depend on the scale of the problem and the resolution of the data, so when remote sensing data are used, it is important to choose the correct sensor according to the objectives. This source of data is highlighted as in the field of ecoepidemiology, and the study of the impact of environmental changes on human health, the effects of land use, and land-cover changes is an increasing area of interest. In addition, these tools associated with the use of algorithms allow modeling and predictions based on different environmental variables like indicators [12–18].

Therefore, the research goal of this paper is to offer examples of applications of objective spatial-based tools, in order to assist in the selection and interpretation of the scales of analysis, describing different studies from diverse scenarios of transmission of leishmaniases in Argentina.

## 2. Materials and Methods

In this work, spatial and temporal scales were used of increasing size and successively inclusive, and consequently, the results are presented in the different scales of analysis: microscale (microfocal), mesoscale (locality or epidemic focus), and macroscale (regional).

### 2.1. Study Area

**Figure 1:**
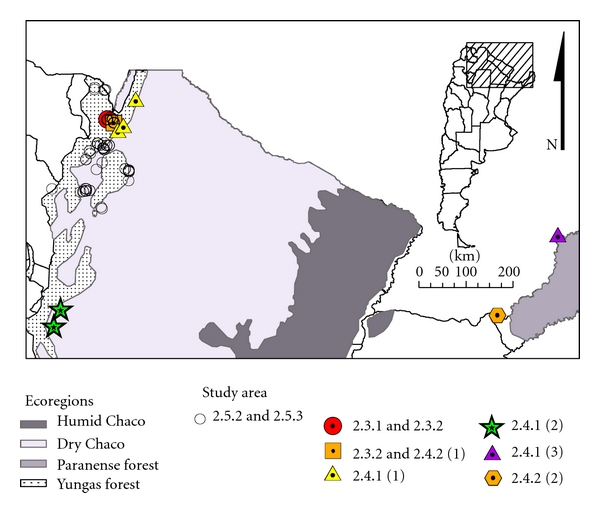
Areas of study in Argentina.

The researches were carried out in an endemic area for TL or VL, and the sites corresponded to three major ecoregions: the Yungas subtropical forest, dry and humid Chaco, and Paranaense forest (Figure 1).

Traps were usually located in the “worst scenario,” an operational definition for the site within the study area, most likely to find phlebotomine due to habitat conditions. Thus, in the context of these designs for spatial analysis and environmental-driven changes in population abundance of vectors, it has more biological significance than a spatial centroid [19, 20]. The specific place and habitat selected in each study depends on the specific objectives of each one (detailed in the results). 

### 2.2. Sampling of Phlebotomine

In general, the insects were captured from afternoon to the following morning by light CDC traps (from 17.00 to 9.00 h) [21]. The sampling effort is detailed in subsequent items for each stage and area of study. In the laboratory, phlebotominae were separated from other insects and then were clarified and mounted for identification. The determination to species level according was made with the key of Young and Duncan [4], modified by Filho et al. [22] to *Lu. neivai*.

### 2.3. Microfocal Scale

Two studies are developed for microscale analysis: the first addressed the problem of “edge effect,” and it was aimed to define the dynamics and distribution of vectors of TL in an area soon to be deforested; the second work was related to the study of the distribution of risk associated with vector abundance and the characterization of “worst scenarios.”

#### 2.3.1. Edge Effect

**Figure 2:**
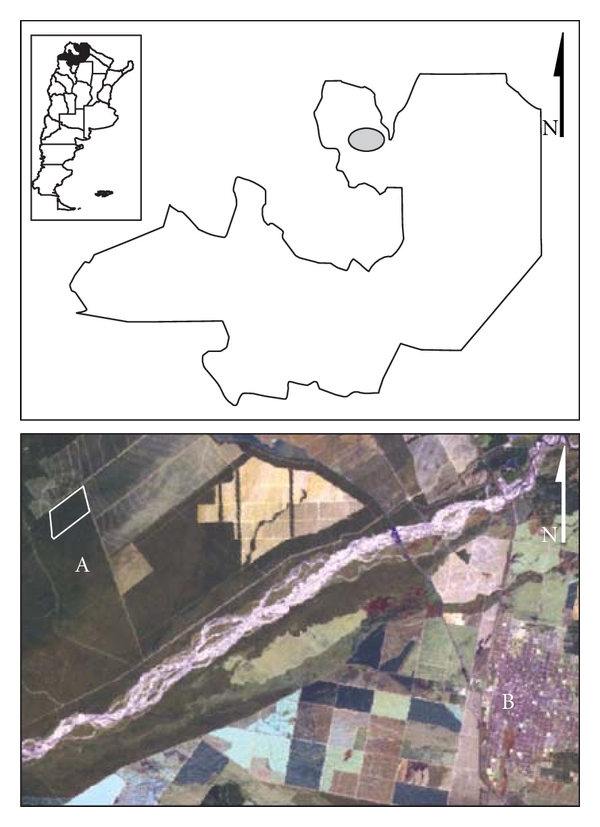
Study area. Landsat 5TM satellite image (231/076, composition bands 752, 18/09/2006), provided by National Commission on Space Activities (CONAE).

In the last two decades, it was speculated about the effect that deforestation has on vector-borne diseases. Thus, in order to evaluate whether “deforestation” influences the dynamics and distribution of TL vectors, an experimental sampling design in an area prone to be deforestated was developed. The deforestation is associated to crop culture highly technified, with regular procedures along the year, and without human settlements, so the deforestation process-induced interface was assumed as the main anthropical-driven intervention in the area [23]. The study area was in northern Argentina, in the hyperendemic area of TL produced by *Le. braziliensis*, and corresponds to the ecoregion of the Yungas subtropical forest (Figure 1). The area had a surface of 1000 × 500 m, where three sets of 5 traps separated by 100 m were placed; two sides of the polygon were in contact with primary vegetation, and the other two sides were in contact with soybean crops (Figure 2). A total of 20 monthly samplings were made including all the seasons. For analysis, we used two secondary matrices, one of them with distance measurements (value in meters from the edge of each trap crop) and the other matrix with the meteorological variables (daily). Twelve meteorological variables were selected based on bibliography of studies made in the same area (minimum, maximum, and mean temperatures [°C]; total precipitation [mm]; mean relative humidity [%]; atmospheric pressure [mbar]; mean visibility [km]; mean wind speed [km/h]; maximum sustained wind speed [km/h]; total number of rainy days per month; total number of stormy days per month; total number of foggy days per month) and were provided by a meteorological station located 4.3 km from the study area, and the Oran Aero station located 25 km from there. To estimate the community of species and the influence of meteorological variables, nonmetric multidimensional scaling (NMDS) and Kendall's correlation coefficients are used. The multivariate analyses were made using PC-ORD 5.0 [24] and univariate analyses [25]. All statistical tests were considered significant at *P* < 0.05. 

#### 2.3.2. Risk Distribution/Abundance/“Worst Scenario”

To assess the distribution of risk at microscale, traps were placed in periurban areas in all the possible shelters and sources of blood intake for the vectors in the *Le. braziliensis* in the TL hyperendemic area [26]. The study was performed in the city of Oran distant 22 km from the study area of point 1 (Figure 2). Oran is the most populous city in the area and has the highest incidence of TL in Argentina. The captures were performed during the season of transmission (five consecutive days) and in the other season of vector activity (four consecutive days). 

The traps were placed on sites considered “worst scenario,” and five variables related to rainfall and temperature were considered (accumulated precipitation, maximum and minimum temperature, night sample without precipitation, and night with drizzles). Meteorological data were obtained from the National Weather Service and Oran Aero station. Test of independence was performed, in order to determine whether or not there are significant differences between the sampling sites based on the abundance of different species, proportion of females/males, and the percentage of pregnant females. Chi-square test was performed, taking the abundance of vector species or its females as an indicator of the distribution of the spatial probability of human-vector contact during interepidemic periods. All statistical tests were considered significant at *P* < 0.05.

Sex ratios (female : male) were computed only when the total capture had more than 15 individuals.

### 2.4. Focal Scale

The mesoscale section presents four works related to landscape changes caused by deforestation, fragmentation, and urbanization. The space-time pattern of phlebotomine is studied during the inter-epidemic period or during the period of increasing human cases of TL or VL in urbanized areas, associated with environmental variables. These spatial-based studies are focused on the vector abundance as indicator of risk due to vector-human effective contacts distributed at foci scale. To have a more appropriate approach to the actual transmission scenario, the infection rate and density of vectors should also be taken into account, but during inter-epidemic periods, the infection is usually very low, besides the fact that the trap-associated captures are representative of a very small area, so the data interpretation of density or infection rates could have microfocal-based biases at mesofocal scale.

#### 2.4.1. Landscape Modification/Deforestation/Fragmentation

(1) The study area corresponds to three municipalities in the northern province of Salta (Pichanal, Embarcación, and Mosconi) (Figure 1) and corresponds to the ecoregions of Yungas and areas of transition to dry Chaco region [27]. The sites are categorized as primary forest, secondary forest, xeric woodlands, periurban, and rural areas. To know the space-temporal pattern of Phlebotominae during a TL inter-epidemic period, captures were made in different environments [28]. The distribution of phlebotomine abundance between the categories of the environment was tested (chi-square).

Three captures were made in each site between October and November. Time series analysis was performed additionally, with the data from one rural and one periurban site in each municipality (the one with more phlebotominae), once a week (year 1), or once every two weeks (year 2) for a 130-week period beginning in October. The meteorological variables (rainfall, mean temperature) were obtained from Cargill SA and station Oran Aero. All statistical tests were considered significant at *P* < 0.01.

 The abundance of phlebotomine was estimated using the William's geometric mean. Time series statistics were computed by the SYSTAT software package [29]. Autoregressive integrated moving average (ARIMA) models and multivariate general linear stepwise models (*P* = 0.15 including covariance) were tested [29, 30].

(2) After an increase of reported cases of TL in Alberdi, Tucumán province, the distribution of phlebotomine and cases was studied in a cross-sectional survey [31]. The study focus was made in the city of Alberdi and surroundings (Figure 1). The collections were made at 10 sites with previous reported cases; the traps were placed overnight on two consecutive nights. The sites were classified according to its potential habitats for phlebotomine in an empirical scale from 1 to 4, which was obtained by adding the following dichotomous attributes (presence 1, absence 0): (a) river or stream at a distance <200 m; (b) land of about 5 m, surface shaded >70%; (c) patches of trees of at least 15 m wide and 5 m in length or a dense patch of sugar cane of 2 m diameter; (d) dwelling or rest place for domestic animals (pigs, goats, horses, and chickens) or a site with minimal disturbance of the vegetation of 50 m². Differences between categories were evaluated with Fisher's exact (with smaller samples) and Chi-square tests. All statistical tests were considered significant at *P* < 0.05.

Meteorological data were obtained from the National Weather Service, Argentina Air Force, and a station from Tucumán capital. The sequential satellite images (Landsat 7 TM) were obtained from the National Commission on Space Activities (CONAE). The case records were obtained from the Hospital of Concepcion and the Provincial Health System of the province of Tucumán (SIPROSA).

During the year following the outbreak described previously, it was reported again the increase of cases of TL in the two nearest (northern) departments of the department of Alberdi, Simoca, and Monteros, in the province of Tucuman [32]. The captures of phlebotomine were made in 29 sites with antecedent of recent cases of TL in the two above-mentioned departments (Figure 1). The environments were characterized as residual vegetation and periurban-vegetated peridomestic habitats related to TL cases. The differences between sites were tested with the same statistical tests used in the previous cross-sectional study. The case records were obtained from the Hospital of Concepcion and the SIPROSA.

(3) The work was realized in a recently deforested area in Iguazú, province of Misiones (Figure 1), where farms of approximately 100 × 400 m were made on the edge of patches of remnant primary or secondary forest. There, the traps were placed in homes and animal pens of three farms from June 2006 to March 2008, with an average periodicity of 15.6 days [33]. The mean abundance (Wilcoxon test) and the composition of the community (test of independence) were compared in homes and in the pens. To study the association between the abundance obtained in each environment in each sample, a Spearman correlation analysis was performed on the average of the three farms and for each farm individually. The association (Spearman) between the abundance of the most abundant species and environmental variables (minimum temperature, daily average, and maximum daily rainfall) was studied. This analysis was done on the abundance obtained at the day of sampling and applying a delay up to 10 samples (156 days). All statistical tests were considered significant at *P* < 0.05.

#### 2.4.2. Urbanization

(1) In the study area, the community had a perception of urban transmission although the biological antecedents (Section 2.4.1 (1)) suggested a different pattern, an usual misperception also observed in other foci [34]. In order to solve this contradiction, captures were performed to assess the relative abundance of vectors in different habitats, as an indicator of the spatial distribution of the probability of human-vector contact during the inter-epidemic period in the urban surrounding [26]. *Lutzomyia* captures were made in the city of Oran (Figure 1) (previously described in point 2) and its surroundings during the two stations of highest activity of phlebotomine (Fall and Spring) in 12 sites identified as “worst scenario” [19, 20]. The variables selected were accumulated precipitation, maximum and minimum temperature, night sample without precipitation, and night with drizzles, obtained from the National Weather Service and Oran Aero stations. To determine differences between the sampling sites, chi-square tests were performed based on the abundance of species, proportion of females/males, and the percentage of pregnant females. All statistical tests were considered significant at *P* < 0.05.

(2) In order to study the spatial distribution of *Lu. longipalpis* in the city of Posadas-Garupá in northeastern Argentina (Figure 1), just after the first autochthonous case of VL was reported, the city was divided into areas of 400 × 400 m [35]. Each trap was placed in a peridomestic habitat, with the criteria for “worst scenario” in each area [19, 20]. To study the spatial dependence of the abundance of *Lu. Longipalpis,* a semivariogram using the spherical function was performed. The abundance of *Lu. longipalpis* was interpolated for the whole study area from the sampling points, using a common *Krigging* procedure [36–39].

A total of 35 environmental variables were selected based on vector biology and variables associated with the risk of VL reported in the literature [40–45] for the 305 sampled homes, and its possible role was evaluated as indicator of the abundance of *Lu. longipalpis*. The environmental variables were obtained based on mapping (tree coverage and water courses), census variables (demographic and socioeconomic level indicators, with resolution of census tract, INDEC 2001) and surveyed at the point of sampling (presence of chickens). A chi-square test was performed for the presence of chickens in houses with and without phlebotomine: (1) considering all dwellings with at least one sand fly captured and (2) considering only those households with medium and high abundance (>30 sand flies). In order to analyze the association of *Lu. longipalpis* with environmental variables, multiple regression analysis, stepwise forward [46] using generalized linear models (GLMs), was performed [47, 48]. All statistical tests were considered significant at *P* < 0.05.

Samplings were repeated in 2009 in order to compare the spatial distribution of the vector between 2007 and 2009. The methodology used for spatial dependence and interpolated abundance of *Lu. longipalpis* was the same as described in the paragraph above. The total surface occupied by areas of medium and low abundance between years (>60 individuals and <30–<60 individuals, resp.) was computed. Although the environmental analysis methodology for 2007 and 2009 samples was the same, the environmental variables in the last year were surveyed at microscale level (35 environmental variables for each sampled house) [49].

### 2.5. Regional Scale

The macroscale research about the distribution of phlebotomines in areas of endemic transmission was performed developing qualitative risk maps and models of potential distributions.

#### 2.5.1. Distribution of Phlebotominae in Endemic Area/Prevalence of Different Species

To develop phlebotomine species presence/absence and relative abundance maps, all published and unpublished records obtained by researchers of the Research Network of Leishmaniasis in Argentina (REDILA) [50], in which the authors are members, were used. The epidemic outbreaks reported in Argentina since 1985 were incorporated on the ecoregions maps according to the place and year of the main focus.  Table 1 shows the records of Phlebotominae present until today in Argentina [10]. *Lutzomyia longipalpis* was recorded since 2004 in the province of Formosa [51].

#### 2.5.2. Qualitative Risk Maps

A risk map was developed from captures made in the provinces of Jujuy and Salta (Figure 1) [56]. To assess differences in species diversity and their abundance were used Fisher'exact (sample sizes small) and chi-quadrate tests. All statistical tests were considered significant at *P* < 0.05.

Categories were defined with different risks of transmission, based on the abundance and diversity of phlebotomine captured.

#### 2.5.3. Maps of Potential Distribution

In the endemic TL area, in northwestern Argentina, the potential distributions of *Lu. neivai* and *Lu. migonei* [56] were modeled from previous records. The analysis of ecological niche modeling was performed with the algorithm of maximum entropy distribution, MaxEnt (maximum entropy modelling system) program. The study included 19 bioclimatic variables (http://www.worldclim.org/) with a resolution of 30 arc-seconds, and each cell is in fact a square of approximately 1 kilometer side (0.93 × 0.93 = 0.86 km^2^). The same resolution was used for the digital elevation model (Shuttle Radar Topographic Mission-SRTM, http://glcf.umiacs.umd.edu/data/srtm/). Jackknife tests were conducted to see which variables are more influential in building models. The model was evaluated with the values of the ROC (receiver operating characteristic) and validated the models with the Kappa index. At the country level, it was modeled on a preliminary distribution of the vector incriminated in the transmission of *Le. infantum*-VL and *Lu. longipalpis*. 

## 3. Results and Discussion

### 3.1. Microfocal Scale

#### 3.1.1. Edge Effect

The impact of human intervention in populations of vectors of *Leishmania* in a scenario of recent deforestation [23] was observed. This hypothesis of increased risk of transmission associated with deforestation was raised also for malaria, Chagas' disease, and other pathologies [57, 58]. *Lutzomyia neivai * had been previously incriminated as the vector in the area of study (TL) and was the most abundant species [59]. The species abundance was associated most frequently with precipitation, and therefore, with humidity, and presence/absence of rain during the sampling dates. Although some association with maximum temperatures was found, this factor would not by itself be able to explain the changes in abundance (Table 2).

The Kendall correlation analysis showed a significant negative strong association between the distance from each trap to the edge of the crop and axis 1 (Kendall Tau = 0.48, *N* = 15, *P* < 0.01). Thus, the traps that were located on the edge of primary vegetation-crop cultures captured a significantly greater number of vectors (mainly *Lu. neivai*) than the traps placed in less disturbed areas. Further, it was found that small modifications of the landscape (deforestation logging) produce an immediate increase in abundance of phlebotomine. The increase in vector abundance occurred at the interface regardless of the presence of human settlements close to the primary vegetation patch, perhaps due to accumulation of potential food sources (granivorous, synanthropic rodents, and richness of niches in the ecotone). The supply of food can make even that occasional wild reservoirs visit peridomestic environment, as observed with sloths [60]. But this effect is in turn magnified when the man is installed with its pets close to the interface [61] then generating a source of permanent shelter and food for the phlebotomine concentrate on the interface.

#### 3.1.2. Risk Distribution/Abundance/“Worst Scenario” 

In this microfocal study 22 km from the area studied in Section 1, in the periurban-ruralized border of Oran city, significant differences were observed in phlebotomine abundance according to the distance between the capture site and the nearest patch of dense vegetation (extradomestic), and between the trap and the distance to the site where an eventual food source or shelter for vector resting was concentrated. The range of simultaneous captures varies from 1 to 3000 *Lu. neivai* individuals between sites 50 m apart from each other (Table 3). In addition to the spatial variation, the variation between days showed that temporary shelters, located between source populations of vectors and the food source on the track of the odor plume of attraction, can hold numerous phlebotomine if animals occasionally overnight there or the weather conditions are adverse to the blood-seeking flight of *Lu. neivai*. Further, the abundance of sand flies appears to be more sensitive to the variable climate in peridomestic habitats than in patches of secondary vegetation. Thus, the heterogeneity of vector abundance in time and space on a small scale should be taken into account in assessing the representativeness of sampling monitoring according to the habitat microheterogeneity [62]. 

### 3.2. Focal Scale 

#### 3.2.1. Landscape Modification/Deforestation/Fragmentation

**Figure 3:**
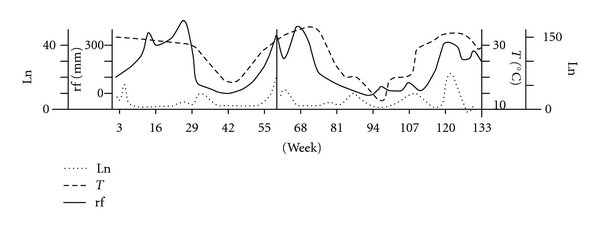
“Lowess” smoothed series *Lutzomyia neivai *captured in Salta, Argentina. Weekly (left, year 1) or every other week (right, year 2) collections. Rainfall (rf) and mean temperature (T) records during the same period.

**Figure 4:**
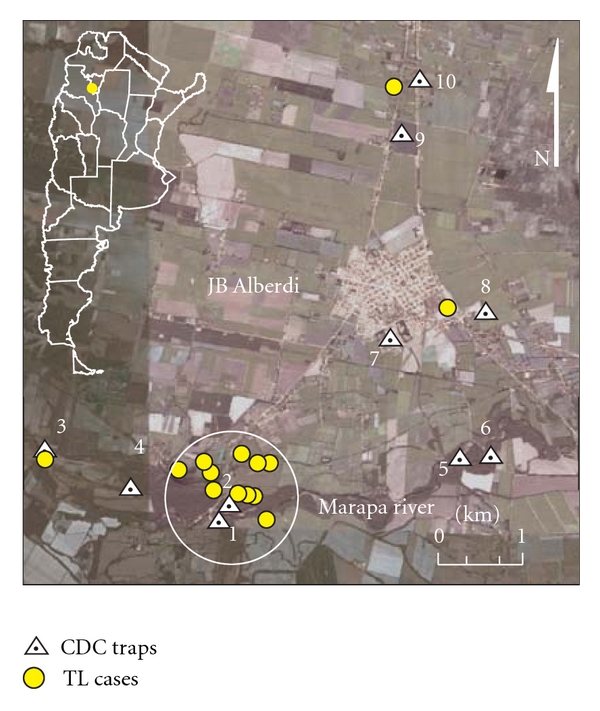
Study area with location of cases and the sites of capture Phlebotominae, Alberdi, Tucumán. The white circle (2 km) encloses the clustered cases and the most abundant sites with phlebotomine. Image taken from Google Earth, version 5.1.3533.1731 http://earth.google.com/.

**Figure 5:**
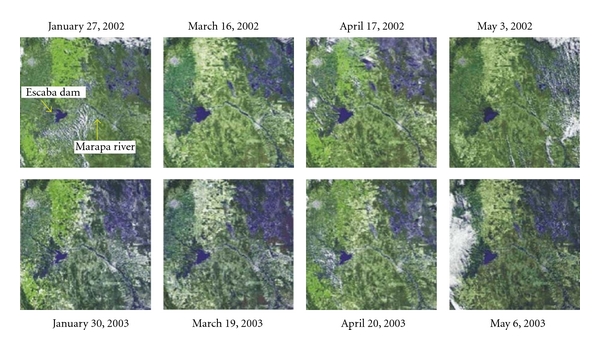
Sequential satellite images from January to March, 2002 and 2003, Alberdi, Tucumán. S103 LANDSAT 7 TM 230/79: 26°29′23′S, 65°19′48′ W, 26°46′12′S, 65°25′ 11′ W, 28°06′36′S, 65°45′00′W, 26°23′23′S, 65°48′36′W; provided by CONAE.

(1) In the hyperendemic area of northwestern Argentina, the results of the longitudinal capture (130 weeks, 45,000 phlebotomines) during the inter-epidemic period just after the first recorded TL outbreak suggested a metapopulation structure dynamics of phlebotomine, by a time series analysis, which found that populations in the patch of vegetation near the homes had a significant autocorrelation every 5-6 weeks (adult-adult cycle). In longer periods (double), the extradomestic population had cross-correlation with the peridomestic population, so periodic settlements of local extinguishable populations (peridomestic) from source populations (extradomestic) were proposed. The vector *Lu. neivai* [59] showed a positive association with rainfall in previous years, due to the generation of new eventual breeding sites and also showed an association with temperature and relative humidity at 20 weeks (increased metabolic activity) (Figure 3). Thus, outbreaks can be caused by unusual rainy periods (ENSO), followed by years of moderate rainfall and temperature [63]. 

(2) The cases of TL in the area of JB Alberdi were clustered in time and space during outbreaks. The main transmission period of TL took place during the first half of the fall. The distribution by age and sex of the cases suggested peridomestic transmission. The spatial distribution of cases showed a strong association of risk with the gallery forest of the Marapa River. The abundance of *Lu. neivai* increased consistently with the scale of habitat type, and so was also consistent with metapopulation dynamics structure (Figure 4). Besides, positive association was observed a between the pattern of rainfall and occurrence of cases, this pattern also coincided with the volume of water observed in sequential images of a nearby dam and detected a minor contribution of the band in the green, which is associated with change in land use or less plant growth (Figure 5). Further, in a close dam area (potential source population), a significant decrease in the population of the colony of insectivore's bats, blood supply to the phlebotomine was produced, so this could have affected the dispersion of vectors. 

 In the captures performed in Simoca and Monteros departments, the most abundant species were *Lu. neivai* (57%) and *Lu. migonei* (42%) (Table 4). *Lutzomyia migonei* is primarily zoophilic and could act as a “hinge” between the zoonotic cycle and the human cases or maintain the parasite circulating in the interepidemic zoonotic cycle [64]. The foci generated during 2004 north to JB Alberdi focus showed similar characteristics in relation to the cases and the composition of Phlebotominae populations more than the mentioned JB Alberdi outbreak described in the previous paragraph, but with a distribution of vectors in space, almost exclusively peridomestic, and a broader temporal and spatial profile of cases. 

(3) In the northeastern country border, close to the Iguazu waterfalls, in a recent deforested area, more than 20.000 Phleblotominae belonging to 17 species were captured associated to the TL focus. *Lutzomyia whitmani* and *Lu. migonei* represent almost the total of the captures in both environments, and *Lu. whitmani* was the dominant species followed by *Lu. migonei*. The abundance of phlebotomine was higher in pigsties than in houses (*P* < 0.0001, 91.26 % in pigsties). *Lutzomyia whitmani* was also the dominant species (up to 98.9%) in many foci of TL in Brazil, where it was suggested to be in the process of adapting to modified environments, present in domestic (endophilic and endophagic) and peridomestic habitats, and feeding on humans, domestic and synanthropic animals [65–71]. This species was present in pigsties and houses during all the seasons, although higher abundances were observed during the warm months. The mean abundance recorded in pigsties and houses was positively associated between these sites for *Lu. Whitmani*, as it was already described [67, 72]. The species is anthropophilic but also an opportunistic feeder, and so the pigsties and hen houses could provide blood, shelter, and breeding places for this vector [73–75]. In both environments, the abundances of *Lu. whitmani* and *Lu. migonei* were associated positively (*P* < 0.05 in both cases) with all the temperature variables when no delay was applied in the analysis, and this may be related to the increase of metabolism of adults. In general, this association reaches a maximum value at 31 or 47 days previous to the captures (increase of metabolism in all the stages), and it was associated positively with the daily accumulated precipitation at 31 days previous to the sampling session, but this association become in significant when the effect of the temperature variables was removed (*P* > 0.05). The precipitation may be related with the humidity of the soil and the survival of the larvae, as the lag is consistent with the phlebotomine larval period [76, 77]. However, probably precipitations are not a limiting factor in this study area, where the climate is subtropical without dry season [78], and the rains are relatively frequent along the year. 

#### 3.2.2. Urbanization

**Figure 6:**
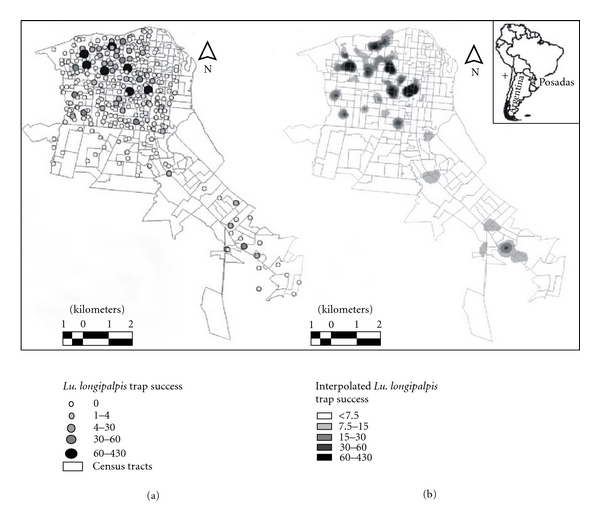
Spatial distribution of *Lu. longipalpis* in Posadas and Garupá. (a) Captures success and (b) predicted abundance interpolated by common *Krigging* procedure.

(1) The captures made around and within Oran city, in the hyperendemeic TL area, showed that *Lu. neivai* was the most abundant species. In Autumn, the most important site in relation with the abundance was associated with animal dwellings with vegetation in the periurban, south-east corner border of the city, followed in abundance by a place with a smaller animal dwelling in the east edge, and two sites with secondary vegetation and an artificial water reservoir in the southern border of the city. In Spring, the site with highest capture rates of *Lu. neivai* was placed on the border of a neighborhood recently settled, again showing an association between the density of vegetation and the abundance of phlebotomine. Thus, there was a distribution of vectors clustered in “hot spots” outside the city, with very few individuals present in only one of five sites within the city. Therefore, as it was previously reported for *Lu. intermedia* in Rio de Janeiro [79], this distribution suggests that the risk of effective human-parasite-vector contact in urban environments during inter-epidemic periods is still associated with patches of periurban vegetation despite the urban residence cases and the perception of urban transmission by the population, even the health system agents. 

(2) After the first autochthonous reported case of human VL in Argentina in Posadas city area, *Lu. longipalpis* was found in the whole Posadas city and the contiguous Garupá village (Figure 1). A total of 2,428 individuals (male/female: 3.5) were captured in 42% of sites sampled (Figure 6(a)). The mean abundance was 8.29 individuals/trap (SD 39.84, range 0:498). In addition, 8 *Lu. cortelezzii*, 3 *Lu. neivai,* and 1 *Lu. whitmani* were captured. *Lutzomyia longipalpis* showed spatial autocorrelation of 590 meters (nugget: 21 mts; maximum variance: 1893) between sites. These parameters showed no change in semivariograms to four different directions, showing isotropy. *Lutzomyia longipalpis* showed an increase in abundance towards the center of the city of Posadas, with six areas of highest abundance (>60 individuals) and other points of mean abundance (30 to 60 individuals) (Figure 6(b)). The best generalized linear model found to explain the abundance of *Lu. longipalpis* included as explanatory variables, the percentage of households with economic deprivation and materials (negative) the percentage of land area covered by trees and shrubs, and percentage of households without electricity (positive). The model explained 31.24% of the total deviance, with 293 degrees of freedom. There was a marginal association between the presences of *Lu. longipalpis* and chickens (*P* = 0.07, odd ratio: 1.53, IC_95_: 95:0.97 : 2.44). However, when considering only positive houses with medium or high abundance (>30 individuals) and the rest as negative, this association was significant (*P* = 0.02, odds ratio: 3.26, IC_95_: 1.217 : 8.77). 

The spatial autocorrelation found in the same study area for 2009 was 688 m, a distance similar to that found in 2007. The pattern of areas of medium and highest abundance in a matrix of low abundance was also preserved. This result confirms the observations made previously in 2007, when the existence of microsites for the establishment of the vector in the urban environment was recorded, consistently with the heterogeneity provided by the urban landscape. This pattern of clustering of areas with high risk of transmission of VL was observed also in Brazil, based on human and canine cases of VL [80, 81]. The mean of captures was three times higher than these obtained in 2007 although the percentage of houses with *Lu. longipalpis* decreased from 2007 to 2009. Regarding the spatial pattern observed in 2009, from the areas of high and medium abundance obtained in 2007, three increased their size and only four of them maintained their position. The area occupied by patches of intermediate and high abundance increased by 6 orders of magnitude from 2007 to 2009. The environmental variables that best explained the abundance of *Lu. longipalpis* to microscale level were the proportion of area occupied by trees and shrubs within a ratio of 100 m from the sampling point, the presence of accumulated junk, cement in peridomicile, and lemon trees. The presence of cement in the peridomicile would indicate that the houses are in the urban area of the city and not in the rural periphery. The proportion of trees and shrubs in a radius of 100 m and the presence of lemon trees and junk in the peridomicile would indicate particular features for insect breeding success as shadow and available organic matter in soil and also exclude the downtown area [82]. 

### 3.3. Regional Scale

#### 3.3.1. Distribution of Phlebotominae in the Endemic Area/Prevalence of Different Species

**Figure 7:**
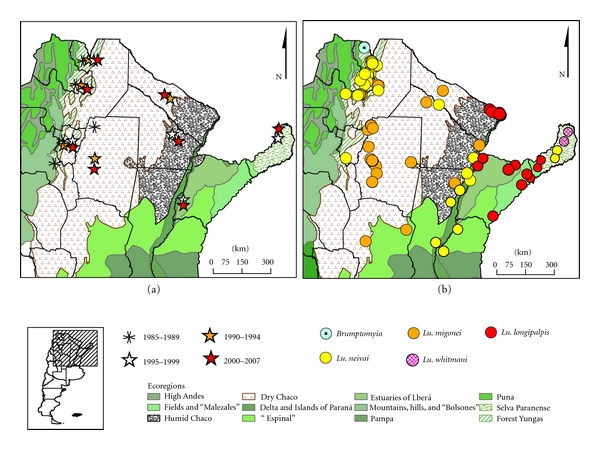
Leishmaniasis in Argentina by ecoregions. Distribution of outbreaks by site and period (a) and distribution of Phlebotominae dominant by different ecoregions with standardized captures (b).* Source of Phlebotominae*: captures of the Research Network of Leishmaniasis in Argentina (REDILA).

The first outbreak of TL in Argentina took place in the northwest of Argentina between 1985 and 1987; from this year until the 90s, there were other outbreaks in the ecoregion of the Yungas. Between 1990 and 1994 besides the outbreaks in the Yungas, the dry Chaco region began to report outbreaks. In the period 1995–1999, the humid Chaco and Paranense region (east) were added to the outbreak records. Finally, from 2000 up to now, human TL cases clustered in time and space in 9 out of the historic 10 endemic provinces are reported periodically as small outbreaks (Figure 7(a)) [1, 10, 50, 83]. Currently, 28 species of phlebotomine were recorded distributed in 13 provinces (Figure 7(b)), and they show a variation in the prevalence (relative abundance) through the ecoregions, consistent with the different epidemiological scenarios.

- Northwest: no transmission and with *Brumptomyia *sp., zoophilic dominant species.
- Yungas forest, humid Chaco, Paranaense región, and gallery forests of larger rivers of the dry Chaco, with suburban/rural outbreaks and *Lu. neivai* dominant, associated with *Lu. migonei*.
- Dry Chaco: with sporadic transmission or clustered by contact with “hot spots” and *Lu. migonei* dominant.

Northern border of Paranaense forest: with outbreaks in deforestations and *Lu. whitmani *as dominant species, this region is the richest in biodiversity. 

#### 3.3.2. Qualitative Risk Maps

**Figure 8:**
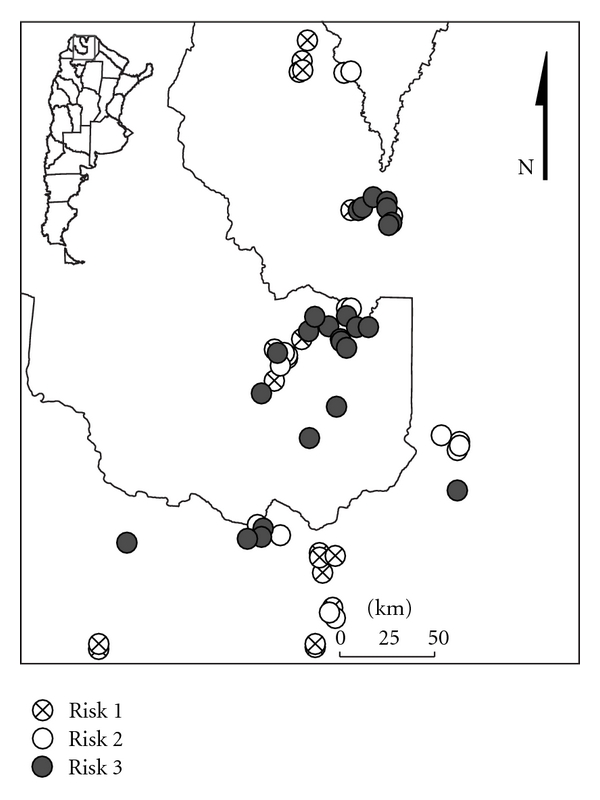
Risk categories by diversity and abundance of Phlebotominae captured in Jujuy and Salta provinces, Argentina. Image taken by Google Earth, version 5.1.3533.1731 http://earth.google.com/.

The risk categories (Figure 8) differed significantly from each other:

- no risk/unknown risk (R1): captures without phlebotomine or with *Brumptomyia *spp. (zoophilic species);
- sporadic transmission risk (R2): with *Lu. cortelezzii*/*Lu. migonei*/*Lu. quinquefer* (not incriminated as vector), wild environments, and cases of TL not much frequent;
- potential transmission risk/epidemic transmission risk (R3): sites with *Lu. neivai* (captures with ≤ 30 phlebotomines/trap) with environmental change, current presence of isolated cases of TL, and sites with *Lu. neivai* (30–3781 phlebotomines captures/trap) + *Lu. migonei* and current presence of clustered cases of TL.

The category R1 was restricted to the area of influence of Baritú National Park in the north of Argentina, the category R2 was recorded in the Chaco region, and finally, the category R3 with potential or actual epidemic risk was mainly located in the Yungas forest, basin of the San Francisco-Bermejo Rivers [56]. 

The last category happens in some areas with low human intervention of the Yungas forest, but mainly in areas heavily modified by man, with constant sources of food for phlebotomine (humans and pets). These categories were consistent with previous data of captures in 14 provinces (>100,000 phlebotomine) [1]. 

#### 3.3.3. Maps of Potential Distribution

The analysis of ecological niche modeling for *Lu. neivai* and *Lu. migonei* resulted in an accuracy close to 1 (perfect [84]) of their geographical distribution. The variable that predicts more effectively the distribution of the data was “the driest month precipitation.” The rainfall influences the reproduction and breeding of this vectors, as it was observed in other studies using different time scales [28, 31, 32, 85], and constituted a significant variance in the dynamics of these insects. The validation of the prediction was more accurate for *Lu. neivai* with a high percentage of sites correctly predicted and a Kappa index with a major agreement with reality [86]. The same analytical approach was used for *Lu. longipalpis* captures of 2009-2010 and 2010-2011, showing that the risk of potential spread of the species is southward along the Uruguay River, on the Argentina-Brazil-Uruguay border (unpublished data). 

## 4. Conclusions

The microscale analysis of the distribution of vectors of TL in northwestern Argentina allowed to verify the edge effect, with an increase of abundance at the interface between primary vegetation and crop, thus indicating one of the mechanisms by which deforestation could increase the risk of human-vector contact, and therefore the risk of transmission of the parasite. Already in the anthropic environment, the distribution and spatial orientation of food sources, physical refuges, and edge with primary vegetation determines the heterogeneous distribution of vectors just in the order of meters. This phenomenon at microscale could explain differences of risk in the nearest and apparently similar scenarios. 

These results also provide evidence to the strategies based on environmental management as an alternative to intervention with insecticides, and also relativize the generalization of data from isolated captures (rates of infection, source of food, abundance, etc.). 

The highest exposure or dependence of phlebotomine to weather conditions in the peridomestic area that in the adjacent natural environments suggests source populations of extradomestic vector, thus the monitoring at domicile/peridomicile reflects better the risk few meters around the capture site, while the monitoring on the edge of primary/secondary vegetation reflects the seasonal pattern of the population. 

Based on these results, new questions were asked for the focal scale, whether the cross-correlations among extradomicile-peridomicile, the clustered distribution in space like “hot spots,” and the clustering in time depending on current and previous weather conditions were consistent with a metapopulation structure. 

According to this metapopulation hypothesis, the source populations of vectors at sites with favorable environmental conditions for feeding and breeding would have the capability to colonize peridomestic sink populations. The landscape changes, the deforestation, fragmentation, and animal management change also the probability of recolonization and stability of the peridomestic populations, concentrating or diluting the vectors, parasites, or reservoirs, by modifications of its source or sink populations. 

In this mesoscale, the climate variables were associated with the success of capture of vectors during the trapping and in previous periods to sampling, according to the modulation of adult activities (food search, attraction to light traps), but also the whole population size in the medium to long-term regulations (metabolic rate, life span, soil conditions for larval stage, etc.). These phenomena were observed for *Lu. neivai* in northwest foci and for *Lu. whitmani* in the northeast focus of Argentina, both with *Lu. migonei* as a possible “hinge” between the zoonotic/anthropo-zoonotic cycle, and with different trends to colonize human environments, according to the history of the focus and the human intervention on the landscape. However, despite this trend toward the domestic habitat, for the species listed, the alleged “urbanization” of the transmission of the etiologic agent of TL in Argentina, from the spatial and temporal study perspective, is still associated with patches of periurban vegetation, in the peridomestic-vegetation ecotone, despite the urban residence of human cases. 

On the other hand, *Lu. longipalpis* is concentrated in hot spots or areas of high and medium abundance, embedded in a matrix of low abundance or absence of vectors within the urban environment. The variables associated with these “hot spots,” by its urban feature are related to attributes of human activities (raising chickens, trees, housing conditions, and landscape of the surrounding public space). This heterogeneous pattern of abundance was maintained between years, despite the increase in human cases, with areas of highest abundance which grew in size, changed position, or generated *de novo*. These changes also suggest the possibility of a metapopulation structure, which changes for or against the reproductive success of the vectors. Further, a “hot spot” could change its size or be extinguished, acting alternatively as a source or sink population. 

The results on the mesoscale support intervention strategies focused in time and space, with higher impact and cost-effectiveness than broader or regular actions against the population of vectors. The mesoscale data also show the difficulty to propose generalized recommendations such as zoo prophylaxis, since the existence of a concentration of food sources close to the man and the consequent increase in vector populations may decrease or increase the risk of human-vector contact. These contradictory outcomes could depend on differences in animal management (colonization of vectors in poultry and pens), the distance, orientation, and size of the source of blood in relation to the site of sleeping of humans, and their habits (housing characteristics, visiting hours and degree of contact with domestic animals, and synanthropic animal attraction sources). 

Based in turn on results in the scale of focus, and the ability of environmental and climatic variables to predict the distribution of vectors, the hypotheses on a regional scale by developing of models based on spatial analysis were raised. 

Maps of historical distribution of outbreaks and of records of vectors were generated; besides, the scenarios of transmission and phlebotomine species present by ecoregion were characterized. The overlap of scenarios and possible vectors allowed in turn develops a qualitative risk map for Argentina. And finally, from the captures data the potential distribution of the main vectors of TL and VL was modeled, defining the climatic variables that best explain the current distribution, and hence the potential distribution if these conditions change. 

These studies allow a better allocation of resources, characterization of scenarios, and defining the appropriate actions in relation to human cases and reservoir (dogs-VL), according to the appearance in the area of epidemic transmission, sporadic cases, or in an area without vectors. Similarly, they allow us to anticipate by projection areas of eventual risk in space and time due to human intervention and settlement, instability, and local or global climate change. 

In conclusion, spatial and temporal analysis of the distribution and abundance of TL and VL vectors in Argentina, discriminated by scales, can generate information that contributes to design strategies for prevention and control of leishmaniasis. It should be noted that the scales from microfocal to regional, although they are inclusive to each other in increasing order, require questions, resolution, data quality, and different analytical tools to support the conclusions appropriate to each scale. 

## Figures and Tables

**Table 1:** Species of Phlebotominae registered by provinces captured by the researchers from the Research Network of Leishmaniasis in Argentina (REDILA), Argentina.

		Provinces												
N	Species	Buenos Aires	Catamarca	Chaco	Córdoba	Corrientes	Formosa	Jujuy	Misiones	Río Negro	Salta	Santiago del Estero	Santa Fe	Tucumán
* Lutzomyia*														
1	*Lu. alphabetica*								X*					
2	*Lu. auraensis*								X*					
3	*Lu. bianchigalatiae*								X					
4	*Lu. cortelezzii*	X*		X		X	X	X	X		X	X	X	X
5	*Lu evandroi*								X					
6	*Lu. fischeri*					X			X					
7	*Lu. lanei*								X					
8	*Lu. longipalpis*					X	X		X					
9	*Lu.migonei*		X	X	X	X	X	X	X		X	X	X	X
10	*Lu. misionensis*					X			X					
11	*Lu. monticola*					X			X					
12	*Lu. neivai*		X	X		X	X	X	X		X	X	X	X
13	*Lu. oswaldoi*								X					
14	*Lu. pascalei*								X					
15	*Lu. peresi*			X										
16	*Lu. pessoai*			X		X			X					
17	*Lu. punctigeniculata*										X			
18	*Lu. quinquefer*			X					X		X			
19	*Lu. sallesi*			X		X								
20	*Lu. shannoni*			X				X			X			X
21	*Lu. sordellii*			X										
22	*Lu. torensis*			X										
23	*Lu. withmani*					X			X					X

* Brumptomyia*														
24	*Br. avellari*			X		X			X					
25	*Br. brumpti*			X					X					
26	*Br. guimaresi*					X	X	X	X		X			
27	*Br. pintoi*								X			X		X

	*Oligodontomyia *sp*. *													
28	[52]									X				

*Historical references, previous to 1960 [53–55].

**Table 2:** Coefficients of Kendall correlations between the values of dates on the NMDS axes based on species composition and meteorological variables (*n* = 40).

Variable	Axis	Kendall Tau
Maximum temperature	1	0.281**
	2	0.309***
Minimum temperature	1	0.045
	2	0.198
Precipitation	1	−0.377***
	2	−0.124
Atmospheric pressure	1	−0.081
	2	−0.191
Relative humidity	1	−0.256**
	2	−0.113
Mean visibility	1	0.242*
	2	0.321***
Mean wind speed	1	−0.109
	2	0.060
Maximum wind speed	1	0.025
	2	0.060
Rainy day	1	−0.280**
	2	0.035
Foggy day	1	−0.086
	2	−0.052

Note: *P < 0.05, **P > 0.01, and ***P > 0.001.

**Table 3:** *Lutzomyia neivai* (female/male) captured by night and site, Oran, Salta, Argentina.

Sites	Day 1	Day 2	Day 3	Day 4	Day 5	Total *Lutzomyianeivai *	F : M	GF%	Total *Lx *
A1	1901/1084	87/54	1/0	6/10	74/18	2069/1166	1.8	0.9^a^	6/2
A2	232/237	32/31	0/0	1/2	6/3	271/273	1.0	3.0^b^	1/0
A3	293/146	81/157	0/0	7/4	33/12	414/319	1.3	2.7^b^	10/12
A4	164/113	35/53	0/0	4/2	12/8	215/176	1.2	5.6^c^	5/6
A5	23/16	0/1	0/0	1/1	1/2	25/20	1.2	12.0^d^	1/2
A6	35/39	5/4	0/0	0	2/0	42/43	1.0	0^e^	2/3
A7	13/6	2/2	0/0	0	0/1	15/9	1.7	13.3^d^	1/1
A8	2/2	0	0/0	0	0	2/2	na	na	0/0
A9	1/1	3/0	0/0	1/1	1/1	6/3	na	na	0/0
A10	25/38	22/27	0/0	1/2	33/12	81/79	1.0	17.3^f^	1/0

Rain	0.0*	0.2**	3.0*	1.0*	0*				
MT	26.5	20.8	12.6	15.6	18.0				
mT	17.3	18.8	11.7	10.7	12.0				

B1	250/240	55/46	—	296/186		601/472	1.3	12.3	5/6
B2	238/66	36/23	38/29	154/85		466/203	2.3	13.1	7/8
B3	—	—	—	2/0		2/0	na	na	1/0
B4	—	3/13	—	2/0		5/13	na	na	0/0
B5	—	—	—	1/0		1/0	na	na	1/0

rf	0.0	0.0	0.0	0.0					
MT	34.0	26.7	29.5	35.6					
mT	16.1	10.0	10.4	16.9					

A1–A10: sites surrounding the pig pen (season of transmission, Autumn); B1–B5: sites surrounding the banana trees patch (season of vector activity, Spring); F : M: *Lu. neivai* female/male ratio; GF%: *Lu. neivai* proportion of gravid females; rf: cumulative rainfall (mm); MT: maximal temperature (C°); mT: minimal temperature (C°); na: less than 15 individuals; a, b, c, d, e, f: each letter differed significantly from the other with a *P* < 0.05; *: without rain during the night; **: drizzles during the night.

**Table 4:** Phlebotominae discriminated by species and habitat (H), Simoca-Monteros, Tucumán, Argentina.

H	T+/TT	*Lutzomyia neivai *N° (%)	*Lutzomyia migonei* N° (%)	*Lutzomyia cortelezzii* N° (%)	Total
T	18/29	201 (57,1)	147 (41,8)	4 (1,1)	352
RV	3/6	11 (73,3)	4 (26,7)	—	15
PU	15/23	190 (56,4)	143 (42,4)	4 (1,2)	337

T: total per site; RV: residual vegetation; PU: periurban.
